# Differential regulation of protein synthesis in skeletal muscle and liver of neonatal pigs by leucine through an mTORC1-dependent pathway

**DOI:** 10.1186/2049-1891-3-3

**Published:** 2012-02-28

**Authors:** Agus Suryawan, Hanh V Nguyen, Rosemarie D Almonaci, Teresa A Davis

**Affiliations:** 1 Department of Pediatrics, Baylor College of Medicine, United States Department of Agriculture/Agriculture Research Service Children's Nutrition Research Center, 1100 Bates Street, Houston, TX 77030, USA

**Keywords:** leucine, mTORC1, neonatal pigs, rapamycin, skeletal muscle

## Abstract

Neonatal growth is characterized by a high protein synthesis rate that is largely due to an enhanced sensitivity to the postprandial rise in insulin and amino acids, especially leucine. The mechanism of leucine's action *in vivo *is not well understood. In this study, we investigated the effect of leucine infusion on protein synthesis in skeletal muscle and liver of neonatal pigs. To evaluate the mode of action of leucine, we used rapamycin, an inhibitor of mammalian target of rapamycin (mTOR) complex-1 (mTORC1). Overnight-fasted 7-day-old piglets were treated with rapamycin for 1 hour and then infused with leucine (400 μmol·kg^-1^·h^-1^) for 1 hour. Leucine infusion increased the rate of protein synthesis, and ribosomal protein S6 kinase 1 (S6K1) and eukaryotic initiation factor (eIF) 4E-binding protein-1 (4E-BP1) phosphorylation in gastrocnemius and masseter muscles (*P *< 0.05), but not in the liver. The leucine-induced stimulation of protein synthesis and S6K1 and 4E-BP1 phosphorylation were completely blocked by rapamycin, suggesting that leucine action is by an mTORC1-dependent mechanism. Neither leucine nor rapamycin had any effect on the activation of the upstream mTORC1 regulators, AMP-activated protein kinase and protein kinase B, in skeletal muscle or liver. The activation of eIF2α and elongation factor 2 was not affected by leucine or rapamycin, indicating that these two pathways are not limiting steps of leucine-induced protein synthesis. These results suggest that leucine stimulates muscle protein synthesis in neonatal pigs by inducing the activation of mTORC1 and its downstream pathway leading to mRNA translation.

## Introduction

One of the hallmarks of the neonatal period is rapid growth, which is due to a high rate of protein synthesis [1]. We previously showed in neonatal pigs that the high rate of deposition of proteins, especially in skeletal muscle, is in part due to their ability to increase protein synthesis in response to feeding, a response that significantly declines with development [2]. We further demonstrated that the feeding-induced stimulation of protein synthesis in most tissues is independently regulated by insulin and amino acids [3]. Among amino acids, we found that leucine alone can stimulate protein synthesis in neonatal pigs and this effect decreases with development [4,5]. However, the molecular mechanism underlying the effect of leucine on the stimulation of protein synthesis *in vivo *is not completely known.

Mammalian target of rapamycin (mTOR) is a nutrient- and hormone-sensitive kinase that plays a major role in cell metabolism, including protein synthesis [6,7]. The kinase mTOR exists in two structurally and functionally distinct complexes referred to as mTOR complex 1 (mTORC1) and mTORC2 [6]. Considerable evidence indicates that mTORC1 is rapamycin sensitive while mTORC2 is rapamycin insensitive. The main function of mTORC1 is to regulate mRNA translation by directly phosphorylating two down-stream substrates, ribosomal protein S6 kinase 1 (S6K1) and eukaryotic initiation factor (eIF) 4E-binding protein-1 (4E-BP1). S6K1 is a kinase for ribosomal protein S6 (rpS6) and its activation by S6K1 is crucial for mRNA translation. Furthermore, a phosphorylated form of 4E-BP1 releases eIF4E from the inactive eIF4E·4E-BP1 complex, allowing the formation of the active eIF4G·eIF4E complex to participate in translation initiation [8]. The second complex, mTORC2, has been postulated to regulate the activation of protein kinase B (PKB) [6]. The exact mechanisms by which nutrients/amino acids, especially leucine, activate mTORC1 have been partly elucidated using cell culture systems [9]. Studies using cell cultures have identified several upstream components, including PKB and AMP-activated protein kinase (AMPK), which are involved in mTORC1 activation [10,11]. Eukaryotic initiation factor 2 and elongation factor 2 (eEF2) pathways have also been shown to be affected by amino acids/leucine [12,13].

Most of the information regarding leucine action on the mTORC1-dependent stimulation of protein synthesis has been generated using cell culture studies [9,14]. Our previous studies have shown that administration of physiological concentrations of leucine can stimulate protein synthesis in neonatal pigs by enhancing the activation of signaling components leading to mRNA translation [15]. Nevertheless, the molecular mechanism of leucine-dependent activation of mTORC1, resulting in enhanced protein synthesis in the neonate, has not been completely studied. Therefore, our study aimed to determine the molecular mechanism by which leucine modulates mTORC1 activation and protein synthesis in skeletal muscle with different fiber types, represented by gastrocnemius and masseter muscles, and in visceral tissues, represented by the liver, using rapamycin as an mTORC1 blocker. We hypothesized that the leucine-induced increase in protein synthesis in skeletal muscle and liver of neonatal pigs will be completely blocked by rapamycin.

## Materials and methods

### Animals and housing

Multiparous crossbred (Landrace × Yorkshire × Duroc × Hampshire) pregnant sows (Agriculture Headquarters, Texas Dept. of Criminal Justice, Huntsville, TX) were housed in lactation crates in individual environmentally controlled rooms before farrowing. Sows were fed a commercial diet (no. 5084, PMI Feeds, Richmond, IN) and provided water ad libitum. After farrowing, piglets remained with the sow and were given supplemental creep feed. Three days before the experiment, piglets were anesthetized for sterile catheter insertion into a jugular vein and carotid artery. Piglets were then returned to the sow and allowed to suckle freely until being studied at 7 days of age (2.0 ± 0.3 kg). This protocol has been previously described [16] and was approved by the Animal Care and Use Committee of Baylor College of Medicine. Studies were conducted in accordance with the National Research Council's *Guide for the Care and Use of Laboratory Animals*.

### Treatments and infusion

Piglets (n = 23) were fasted for 12 to 14 hours before infusion and placed in a sling restraint system. Pigs were randomly assigned to one of four treatment groups: 1) saline (control), 2) saline + rapamycin, 3) leucine, and 4) leucine + rapamycin. Piglets assigned to the rapamycin groups were injected with a rapamycin solution (0.75 mg/kg in 5% dimethyl sulfoxide) 1 hour before the initiation of the leucine infusion while other pigs were injected with diluent. Leucine infusion was initiated with a priming dose (148 μmol/kg) for 10 minutes, followed by a constant infusion of leucine at 400 μmol·kg^-1^·h^-1 ^for 1 hour. This infusion rate was chosen because our previous studies [15] showed that a two- to three-fold elevation in plasma leucine concentrations, similar to those observed with feeding, was achieved by this rate of leucine infusion. During the priming and constant infusion period, saline-infused pigs received a volume of saline equal to that of those receiving leucine.

### Tissue protein synthesis in vivo

Fractional rates of protein synthesis were measured with a modification of the flooding dose method [2,17]. At 30 minutes before the end of the infusion, pigs were injected with 10 mL/kg body weight of a flooding dose of phenylalanine (Amersham Biosciences, Piscataway, NJ), which provided 1.5 mM phenylalanine/kg body weight and 0.5 mCi L-[4-^3^H]phenylalanine/kg body weight. Samples of whole blood were taken 5, 15, and 30 minutes after the injection for measurement of the specific radioactivity of the extracellular free pool of phenylalanine. Pigs were killed at 60 minutes, and samples of the gastrocnemius and masseter muscles and liver were collected, immediately frozen in liquid nitrogen, and stored at -70°C until later analysis, as previously described [2].

Protein synthesis (*K*_s _expressed as % protein synthesized in a day) was calculated as *K*_s _(%/day) = [(S_b_/S_a_) × (1,440/*t*)] × 100, where S_b _is the specific radioactivity of the protein-bound phenylalanine; S_a _is the specific radioactivity of the tissue free phenylalanine for the labeling period, determined from the value for the animal at the time of tissue collection, and corrected by the linear regression of the blood-specific radioactivity of the animal against time; and *t *is the time of labeling in minutes. Previous studies have demonstrated that after a flooding dose of L-[4-^3^H]phenylalanine is administrated, the specific radioactivity of tissue free phenylalanine is in equilibrium with aminoacyl tRNA specific radioactivity, and therefore, tissue free phenylalanine is a valid measure of the tissue precursor pool specific radioactivity [18].

### Tissue extraction and immunoblot analysis

Tissue samples were homogenized and centrifuged at 10,000 *g *for 10 minutes at 4°C. Supernatants were diluted in sample buffer, frozen in liquid nitrogen, and stored at -70°C until analysis. Equal amounts of protein samples were electrophoretically separated on polyacrylamide gels and transferred to a polyvinylidene difluoride membrane (Bio-Rad, Hercules, CA). The membrane was incubated with appropriate primary antibodies, washed, and exposed to an appropriate secondary antibody as previously described [16].

For normalization, immunoblots performed with anti-phospho-specific antibodies were stripped in stripping buffer (Pierce Biotechnology, Rockford, IL) and reprobed with the corresponding non-phospho-specific antibodies. Blots were developed with an enhanced chemiluminescence kit (Amersham Biosciences, Piscataway, NJ), visualized, and analyzed with a ChemiDoc-It Imaging System (UVP, Upland, CA). Primary antibodies that were used in the immunoblotting were PKB (total and Ser473; Cell Signaling Technology, Beverly, MA), AMPK-α (total and Thr172; Cell Signaling Technology, Beverly, MA), eIF2α (total and Ser51; Cell Signaling Technology, Beverly, MA), S6K1 (total and Thr398; Cell Signaling Technology, Beverly, MA), 4E-BP1 (total; Bethyl Laboratories, Montgomery, TX, and Thr70; Cell Signaling Technology, Beverly, MA), and eEF2 (total and Thr56; Cell Signaling Technology, Beverly, MA).

### Statistics

All data were analyzed using 2 × 2 factorial analysis. When a significant overall effect was observed, differences among individual means were assessed by the Tukey-Kramer comparisons test. Probability values of *P *< 0.05 were considered statistically significant. Data are presented as means ± SEM.

## Results

### Protein synthesis in skeletal muscles and liver

**Figure 1:**
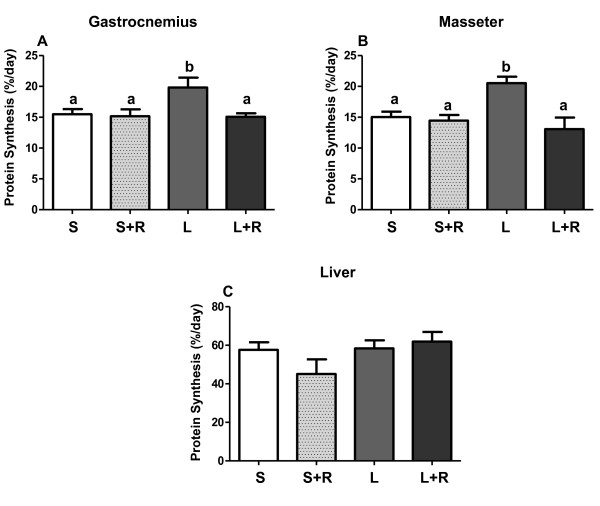
**Fractional rates of protein synthesis in gastrocnemius muscle (A), masseter muscle (B) and liver (C) of 7-day-old pigs after 60 minutes of infusion of saline (Sal), saline with rapamycin (Sal+Rap), 400 μmol·kg^-1^·h^-1 ^leucine without rapamycin (Leu), or 400 μmol·kg^-1^·h^-1 ^leucine with rapamycin (Leu+Rap)**. Values are means ± pooled SE; *n *= 5-7 per treatment. Values with different superscripts differ significantly (*P *< 0.05).

Fractional rates of protein synthesis in the gastrocnemius muscle, which primarily contains glycolytic fibers, and masseter muscle, primarily containing oxidative fibers, were enhanced by leucine infusion at physiological levels (*P *< 0.05; Figure 1). However, leucine had no effect on protein synthesis in the liver. In the current study, we wished to determine whether inhibition of mTORC1 activation by rapamycin could suppress leucine-induced stimulation of protein synthesis in neonatal pigs. We found that rapamycin administration completely blocked leucine-induced protein synthesis in both muscle types (*P *< 0.05; Figure 1). Rapamycin administration had no effect on the basal fasting rate of protein synthesis in all tissues determined.

### Effect of leucine on the activation of signaling components upstream and downstream of mTORC1

**Figure 2:**
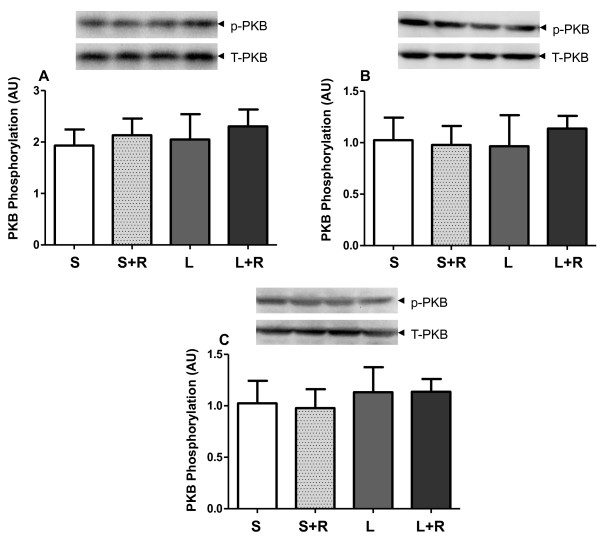
**Phosphorylation of PKB in gastrocnemius muscle (A), masseter muscle (B) and liver (C) of 7-day-old pigs after 60 minutes of infusion of saline (Sal), saline with rapamycin (Sal+Rap), 400 μmol·kg^-1^·h^-1 ^leucine without rapamycin (Leu), or 400 μmol·kg^-1^·h^-1 ^leucine with rapamycin (Leu+Rap)**. Values are means ± pooled SE; *n *= 5-7 per treatment.

**Figure 3:**
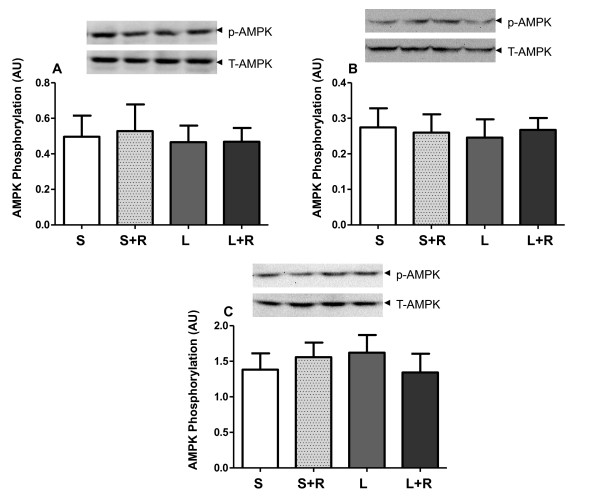
**Phosphorylation of AMPK in gastrocnemius muscle (A), masseter muscle (B) and liver (C) of 7-day-old pigs after 60 minutes of infusion of saline (Sal), saline with rapamycin (Sal+Rap), 400 μmol·kg^-1^·h^-1 ^leucine without rapamycin (Leu), or 400 μmol·kg^-1^·h^-1 ^leucine with rapamycin (Leu+Rap)**. Values are means ± pooled SE; *n *= 5-7 per treatment.

**Figure 4:**
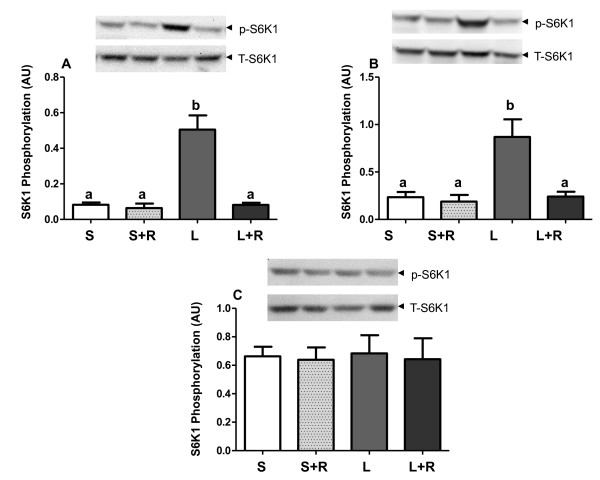
**Phosphorylation of S6K1 in gastrocnemius muscle (A), masseter muscle (B) and liver (C) of 7-day-old pigs after 60 minutes of infusion of saline (Sal), saline with rapamycin (Sal+Rap), 400 μmol·kg^-1^·h^-1 ^leucine without rapamycin (Leu), or 400 μmol·kg^-1^·h^-1 ^leucine with rapamycin (Leu+Rap)**. Values are means ± pooled SE; *n *= 5-7 per treatment. Values with different superscripts differ significantly (*P *< 0.05).

**Figure 5:**
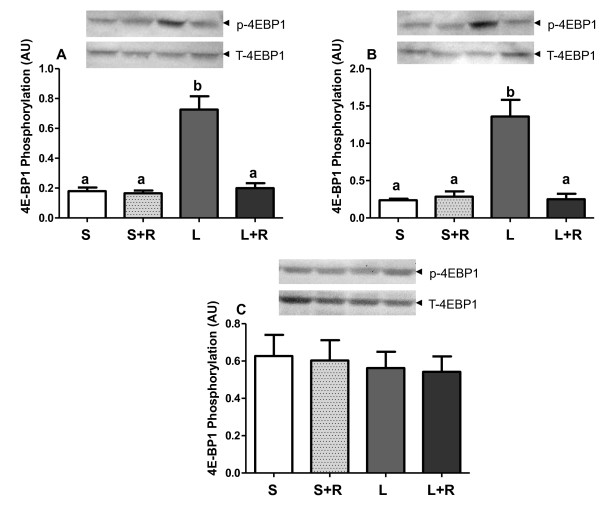
**Phosphorylation of 4E-BP1 in gastrocnemius muscle (A), masseter muscle (B) and liver (C) of 7-day-old pigs after 60 minutes of infusion of saline (Sal), saline with rapamycin (Sal+Rap), 400 μmol·kg^-1^·h^-1 ^leucine without rapamycin (Leu), or 400 μmol·kg^-1^·h^-1 ^leucine with rapamycin (Leu+Rap)**. Values are means ± pooled SE; *n *= 5-7 per treatment. Values with different superscripts differ significantly (*P *< 0.05).

**Figure 6:**
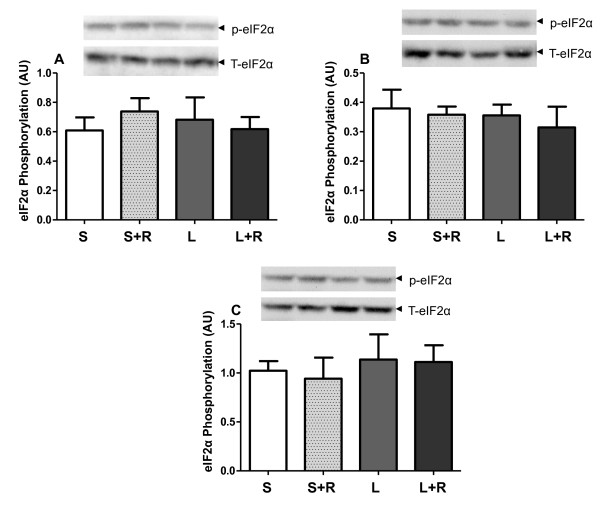
**Phosphorylation of eIF2α in gastrocnemius muscle (A), masseter muscle (B) and liver (C) of 7-day-old pigs after 60 minutes of infusion of saline (Sal), saline with rapamycin (Sal+Rap), 400 μmol·kg^-1^·h^-1 ^leucine without rapamycin (Leu), or 400 μmol·kg^-1^·h^-1 ^leucine with rapamycin (Leu+Rap)**. Values are means ± pooled SE; *n *= 5-7 per treatment.

**Figure 7:**
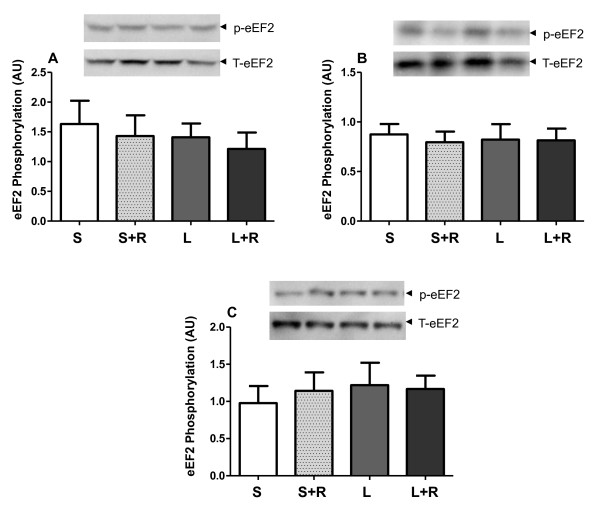
**Phosphorylation of eEF2 in gastrocnemius muscle (A), masseter muscle (B) and liver (C) of 7-day-old pigs after 60 minutes of infusion of saline (Sal), saline with rapamycin (Sal+Rap), 400 μmol·kg^-1^·h^-1 ^leucine without rapamycin (Leu), or 400 μmol·kg^-1^·h^-1 ^leucine with rapamycin (Leu+Rap)**. Values are means ± pooled SE; *n *= 5-7 per treatment.

The molecular mechanisms responsible for the positive effect of leucine on *in vivo *protein synthesis are not completely understood. Therefore, in the current study, we investigated the effect of leucine on the activation of signaling components leading to protein synthesis. We determined the activation of PKB and AMPK (upstream effectors of mTORC1), S6K1 and 4E-BP1 (commonly used as mTORC1 readouts), and eIF2α and eEF2 (signaling factors crucial for translation initiation and elongation, respectively). In gastrocnemius and masseter muscles and liver, leucine infusion had no effect on the phosphorylation of PKB or AMPK (Figures 2 and 3). Leucine infusion robustly induced the phosphorylation of S6K1 and 4E-BP1 in gastrocnemius and masseter muscles, but not in the liver (*P *< 0.05; Figure 4 and 5). Blocking the activation of mTORC1 by rapamycin administration completely inhibited the leucine-induced phosphorylation of S6K1 and 4E-BP1 (*P *< 0.05; Figures 4 and 5). Leucine infusion had no effect on the phosphorylation of eIF2α and eEF2 in all tissues examined (Figures 6 and 7).

## Discussion

Leucine serves as a precursor for protein synthesis and also acts as a nutrient signal that regulates protein synthesis both *in vitro *and *in vivo *[19,20]. A great deal of information regarding the molecular mechanism by which leucine increases protein synthesis has been generated from cell culture studies [9]. However, information on leucine acting in culture may not be applicable to the situation in whole animals because of the different types of cell culture used and the complexity of *in vivo *environments. Therefore, we and others have studied the possible mechanism of the leucine-induced stimulation of protein synthesis using animal models [15,21].

We have previously demonstrated the ability of leucine to stimulate protein synthesis in neonatal pigs through the activation of mTORC1 leading to mRNA translation, but its effect is less than that of a complete amino acid mixture [15]. We found that acute (1 hour) leucine infusion enhanced protein synthesis; however, leucine-induced protein synthesis could not be maintained for 2 hours despite continued mTORC1 activation [15]. This finding was due to the leucine-induced fall in amino acid levels because they were used for protein synthesis [5]. When the fall in amino acids was prevented, the leucine-induced increase in protein synthesis was restored. These findings are an example of the complicated outcomes from *in vivo *studies compared with those from cell culture studies.

Although the leucine-induced activation of mTORC1 is well known, the molecular mechanism underlying this effect is not completely understood [9,22]. A valuable tool for studying mTORC1 activation is the use of rapamycin, a well-characterized mTORC1 inhibitor [23]. In a previous study, we found that rapamycin blocked the feeding-induced stimulation of protein synthesis in the liver but the rate of protein synthesis was only attenuated by 60% in skeletal muscle [24]. In contrast to the previous feeding study [24], in the current study, we did not observe any effect of leucine on liver protein synthesis. One possible explanation for this discrepancy between studies is the different mode of nutrient administration (enteral feeding vs. parenteral infusion). In the current study, in skeletal muscle, rapamycin completely blocked the leucine-induced stimulation of protein synthesis, suggesting that leucine action is solely though the mTORC1 pathway.

PKB and AMPK, upstream signaling components of mTORC1, can positively (PKB) and negatively (AMPK) control mTORC1 activation [10,11]. Our previous research [25] showed that a physiological rise in amino acids *in vivo *has no effect on the phosphorylation of PKB and AMPK in skeletal muscle of neonatal pigs, indicating that amino acid-induced activation of mTORC1 is independent of these signaling components. In the current study, we found that leucine did not stimulate the phosphorylation of PKB in skeletal muscle and liver. Our finding is in agreement with others who showed similar observations in human skeletal muscle [26] and rat skeletal muscle [27]. However, there are conflicting results among studies regarding leucine's effect on AMPK activation. In a cell culture study [28], leucine treatment decreased the phosphorylation of AMPK in C2C12 myoblasts. In contrast, in isolated rat muscle, leucine failed to affect the phosphorylation of AMPK [29]. Our recent *in vivo *study demonstrated that infusion of physiological levels of leucine does not affect the phosphorylation of AMPK in skeletal muscle of neonatal pigs [30]. Similarly, in this study, leucine had no effect on the phosphorylation of AMPK in skeletal muscle and liver.

Genetic and biochemical evidence strongly support that S6K1 and 4E-BP1 play pivotal roles as mTORC1 substrates responsible for stimulating protein synthesis by controlling the step of translation initiation involving the binding of mRNA to 40S ribosomal subunits [8]. In the current study, we found that leucine enhanced the phosphorylation of S6K1 and 4E-BP1 in both skeletal muscles, and that this effect was completely blocked by rapamycin administration. These results clearly indicate that leucine activation of both signaling components is wholly mediated by mTORC1. Consistent with the lack of response of liver protein synthesis, leucine did not affect the phosphorylation of S6K1 and 4E-BP1 in the liver of neonatal pigs.

Amino acids have been shown to be involved in the regulation of the eIF2 pathway, a rate limiting step in translation initiation [12]. In this pathway, eIF2 facilitates the association of initiator methionyl-tRNA_i _(Met-tRNA_i_) with the 40S ribosomal subunit, an absolute requirement for the initiation of translation [12]. Two factors are recognized in this pathway, eIF2α (inhibitor) and eIF2B (activator). Under cell stress, such as amino acid starvation, a kinase for eIF2α, called general control nonrepressed 2 (GCN2), is activated, resulting in the phosphorylation of eIF2α. In the phosphorylated state, eIF2α acts as potent inhibitor of eIF2B. Conversely, under amino acid-rich conditions, GCN2 activation is suppressed. This enables eIF2B to promote the formation of the Met-tRNA_i_-40S ribosomal subunit complex and to initiate mRNA translation (12. In our study, leucine had no effect on the phosphorylation of eIF2α in both the liver and skeletal muscle.

Studies using cell cultures have identified a positive role of leucine in the regulation of the peptide-chain elongation process, which is regulated by the eEF2 pathway [31]. In this pathway, the phosphorylation of eEF2, which results in suppression of the elongation process, is tightly regulated by eEF2 kinase [13]. Insulin or amino acid-induced activation of mTOR/S6K1 signaling causes inactivation of this eEF2 kinase, followed by dephosphorylation of eEF2. Anabolic stimuli decrease eEF2 phosphorylation, enabling the elongation process to occur [14]. Interestingly, in contrast to cell culture studies, our data showed that leucine did not have any effect on phosphorylation of eEF2. This finding suggests that the elongation process is not a rate limiting step in the leucine-induced stimulation of protein synthesis in skeletal muscle of neonatal pigs.

In summary, our study results show that leucine can stimulate protein synthesis in skeletal muscle of neonatal pigs. The inability of leucine to enhance protein synthesis in the liver is probably due to the provision of leucine parenterally, rather than enterally, to the animal. Another possibility is that a higher leucine level is required to induce liver protein synthesis. Our results also strongly support the notion that the leucine-induced stimulation of protein synthesis is mTORC1-dependent, which is consistent with the findings from *in vitro *studies. In conclusion, leucine administration has a potential role in enhancing growth of neonatal animals.

## Competing interests

The authors declare that they have no competing interests.

## Authors' contributions

AS participated in the design of the study and the infusion experiments, performed the statistical analysis, and drafted the manuscript. HVN participated in the infusion experiments and performed the protein synthesis analysis. RDA participated in the infusion experiments and performed the immunoblotting analysis. TAD conceived of the study, participated in its design and coordination, helped to draft the manuscript, and has primary responsibility for the final content. All authors read and approved the final manuscript.
